# A228 SAFETY AND EFFICACY OF ENDOSCOPIC RESECTION OF NON-AMPULLARY DUODENAL POLYPS AND RISK OF POLYP RECURRENCE

**DOI:** 10.1093/jcag/gwac036.228

**Published:** 2023-03-07

**Authors:** Y Hanna, F Dang, S Li, M Kim, J Mosko, G May, C Teshima

**Affiliations:** 1 Division of Gastroenterology and Hepatology, University of Toronto, Toronto; 2 Division of Gastroenterology and Hepatology, University of Calgary, Calgary, Canada; 3 Division of Gastroenterology and Hepatology, St. Vincent's Hospital Sydney, Sydney, Australia; 4 The center for Therapeutic Endoscopy and Endoscopic Oncology, St. Michael's Hospital, Toronto, Canada

## Abstract

**Background:**

Non-ampullary duodenal adenomas, which can present sporadically or in the context of a polyposis syndrome, carry a risk of progression to carcinoma in a similar sequence to colorectal adenomas. Complete endoscopic resection is recommended as first line as a less invasive alternative to surgical rsection. Identifying recurrence rates of non-ampullary duodenal polyps after endoscopic resection, and patient and polyp characteristics associated with recurrence is important in determining the best method of resection and guiding endoscopic surveillance.

**Purpose:**

To determine the technical success rate of endoscopic resection of non-ampullary duodenal polyps, complication rates, rate of residual and recurrent polyps, and identify factors associated with polyp recurrence.

**Method:**

All adult patients (≥18 years) that underwent endoscopic resection of non-ampullary duodenal polyps at St. Michael’s Hospital, a Canadian tertiary referral center, from January 2010 to June 2021 were retrospectively identified. Descriptive statistics were calculated for variables of interest and Chi-square, t-test or U-Mann Whitney tests were used to compare variables as appropriate. Bi-variate regression analysis was utilized to determine co-variables associated with recurrence.

**Result(s):**

A total of 300 patients underwent endoscopic resection of duodenal polyps. Table 1 describes patient demographics, polyp and procedural characteristics and characteristics associated with recurrence. Nearly all cases were technically successful (96%, n=286/299). Clinically significant intraprocedural bleeding occurred in 22% (n=65/300) of patients, and deep mural injury occurred in 3% (n=7/284) of patients. Delayed post-procedural bleeding occurred in 9% of patients (n=26/279). The median time to first surveillance EGD was 4 months. Polyp recurrence occurred in 28% (n=50/180) of patients. Of the patients with polyp recurrence, 82% (n=42/50) were successfully managed endoscopically. On univariate analysis, polyp size (OR 1.03, 95% CI 1.01-1.06), piecemeal resection (OR 1.63, 95% CI 0.17-0.82), intraprocedural bleeding (OR 2.28, 95% CI 1.09-4.74), and high-grade dysplasia (HGD) or intramucosal adenocarcinoma (IMCa) on final histology (OR 3.46, 95% CI 1.64-7.33) were significantly associated with polyp recurrence. On multivariate analysis, only HGD/IMCa on final histology was significant (OR 3.41, 95% CI 1.38-8.47).

**Image:**

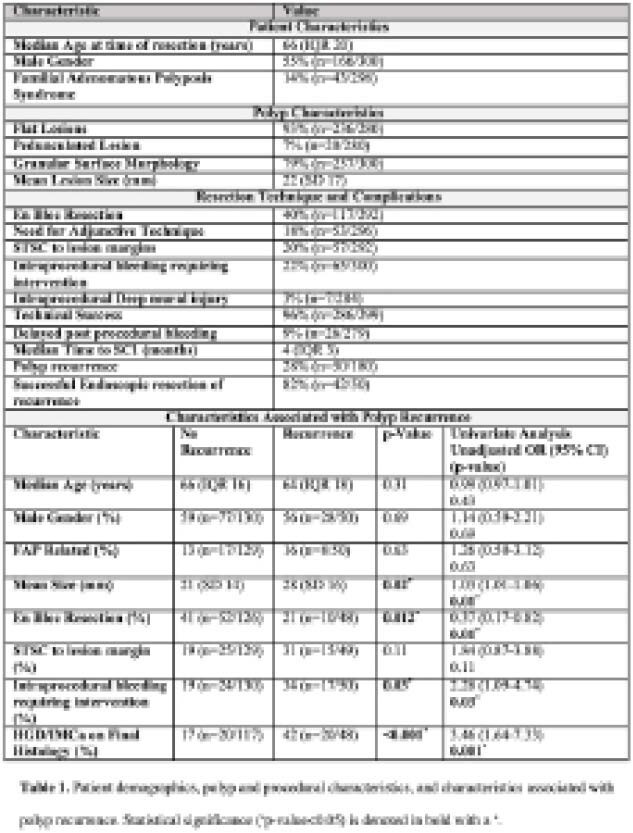

**Conclusion(s):**

Endoscopic resection of duodenal polyps can be safely performed with high technical success, however recurrence is a significant concern. Advanced histology was a significant predictor of polyp recurrence and highlights the importance of accurate pre-resection endoscopic characterization to correctly identify lesions at increased risk that may benefit from alternative resection methods such as ESD or hot rather than cold EMR, and which may require closer follow-up. Future work to develop predictive models of recurrence are needed to better stratify patient risk.

**Please acknowledge all funding agencies by checking the applicable boxes below:**

None

**Disclosure of Interest:**

None Declared

